# Opposition to Inbreeding Between Close Kin Reflects Inclusive Fitness Costs

**DOI:** 10.3389/fpsyg.2018.02101

**Published:** 2018-11-02

**Authors:** Jan Antfolk, Debra Lieberman, Christopher Harju, Anna Albrecht, Andreas Mokros, Pekka Santtila

**Affiliations:** ^1^Department of Psychology, Åbo Akademi University, Turku, Finland; ^2^Department of Psychology, University of Miami, Coral Gables, FL, United States; ^3^Department of Psychology, FernUniversität in Hagen, Hagen, Germany; ^4^Faculty of Arts and Science, New York University Shanghai, Shanghai, China

**Keywords:** inbreeding avoidance, mate choice, inclusive fitness theory, social cognition, inbreeding

## Abstract

Due to the intense selection pressure against inbreeding, humans are expected to possess psychological adaptations that regulate mate choice and avoid inbreeding. From a gene’s-eye perspective, there is little difference in the evolutionary costs between situations where an individual him/herself is participating in inbreeding and inbreeding among other close relatives. The difference is merely quantitative, as fitness can be compromised via both routes. The question is whether humans are sensitive to the direct as well as indirect costs of inbreeding. Using responses from a large population-based sample (27,364 responses from 2,353 participants), we found that human motivations to avoid inbreeding closely track the theoretical costs of inbreeding as predicted by inclusive fitness theory. Participants were asked to select in a forced choice paradigm, which of two acts of inbreeding with actual family members they would want to avoid most. We found that the estimated fitness costs explained 83.6% of participant choices. Importantly, fitness costs explained choices also when the self was not involved. We conclude that humans intuit the indirect fitness costs of mating decisions made by close family members and that psychological inbreeding avoidance mechanisms extend beyond self-regulation.

## Introduction

From a gene’s-eye perspective, there is little evolutionary difference between the costs of selecting fitness-jeopardizing sexual partners oneself and the costs when other family members do the same (Hamilton, 1964). The likelihood that a given allele will be passed onto future generations can be substantially compromised via either route. One mating arrangement that imposes substantial fitness costs is inbreeding between close kin (i.e., sex between closely related biological kin). Given the intense selection pressures posed by short generation pathogens (Hamilton, 1980; Tooby, 1982) and deleterious recessive mutations (Bittles and Neel, 1994; Charlesworth and Charlesworth, 1999), humans (Westermarck, 1891; Lieberman and Antfolk, 2015) and a wide range of other species (Manson and Perry, 1993; Pusey and Wolf, 1996; Bretman et al., 2004; Lemaitre et al., 2012) have evolved systems promoting the avoidance of the fitness costs associated with inbreeding.

Each instance of inbreeding carries multiple types of fitness consequences: There are (i) direct and (ii) indirect fitness consequences to each individual in the inbreeding pair. In addition, there are (iii) indirect fitness consequences to third parties related to the individuals in the inbreeding pair (Dawkins, 1983; Haig, 1999; Antfolk et al., 2012b). Take, for example, an instance of sibling inbreeding. Producing a child that suffers from inbreeding depression (i.e., lowered biological fitness as the result of inbreeding) has a direct effect on the brother’s and, separately, on the sister’s fitness. But the brother’s fitness is also affected indirectly via his sister; he forgoes having a healthy niece or nephew had his sister mated with a non-relative at the price of a more highly related yet less viable child (Adams and Neel, 1967; Kumar et al., 1967; Seemanova, 1971). By analogy, the same holds for the sister. This is true also in other types of inbreeding, such as between a parent and a child or between an uncle or aunt and a niece or nephew.

Extending outward from an inbreeding pair (two related individuals engaging in sex) is a web of related individuals – mother, father, other siblings etc.–each with a unique perspective on the magnitude of costs associated with the siblings producing an inbred child. For this reason, adapted mechanisms that reliably estimate the costs of inbreeding, not only for the purpose of regulating one’s own mating behavior, but also for regulating the mating behavior of close genetic relatives are hypothesized to exist. This generates the testable prediction that for a given event of inbreeding, the possible costs to a focal person’s fitness should map on to the intensity of opposition to the event of inbreeding. That is, by hypothesis, proximate aversions should track estimated ultimate costs. Moreover, this should hold both when individuals assess the costs of inbreeding themselves and the costs of inbreeding between two close relatives.

The fitness costs of a given event of inbreeding for a focal individual (i.e., the person for whom the fitness consequences are calculated) can be estimated as:

(1) $$\delta_{xy}(r_{x}+r_{y})$$

where *r_x_* and *r_y_* signify the relatedness (*r*) between the focal person and individual *X* and individual *Y*, engaging in inbreeding, and δ is the fitness compromise for a given inbred offspring as compared to a non-inbred offspring. The magnitude of δ is determined by the relatedness between the two persons, *X* and *Y*. If, for example, a focal person him/herself engages in inbreeding with a full sibling, the focal person is *X* and the full sibling is *Y*. Because the focal person is 100% related to him/herself, *r_x_* is 1; *r_y_* is 0.5 for the full sibling. If δ_xy_ = 0.3 in offspring from inbreeding full siblings, where *_xy_* = 0.5, this means that the total cost of inbreeding to the focal person is 0.45, that is, 0.3(1 + 0.5) in this case. If, instead, a sibling of the focal person engages in inbreeding with their common sibling, the relatedness between the focal person and both individuals in the inbreeding union is 0.5. In this case, both *r_x_* and *r_y_* are 0.5. Also in this case, the fitness decrease in their offspring is 0.3, but the cost to the focal person is 0.3, that is, 0.3(0.5 + 0.5). As the relatedness between persons *X* and *Y* decreases, the fitness compromise in the inbred offspring also decreases. For example, in the case of inbreeding between cousins δ_xy_ = 0.075.

In general, expression (1) is modeled to be an approximation of the costs associated with a given event of inbreeding. However, the exact relatedness term, *r*, is purely theoretical. Rather than following the theoretical *r*, natural selection will in this case operate through the *perceived* degree of relatedness, that is, an internally estimated, psychological variable. Indeed, prior work on kin detection in humans suggests that humans do not possess exact representations of the true degree of relatedness between two individuals (Apicella and Marlowe, 2004; Lieberman et al., 2007; Tal and Lieberman, 2007; Antfolk et al., 2014b; Billingsley et al., 2018). Humans estimate *r* based on cues that ancestrally correlated with genetic relatedness. Thus, a more exact expression of the costs of inbreeding should weight *r* by the perceived certainty of relatedness–that is, an individual’s psychological estimate of how likely a given family member is a relative and to what degree. This can be expressed by including two kinship certainty (*C*) factors, which weight the *r* by subjective certainty of relatedness to *X* and *Y*, respectively:

δ*_xy_*(C_x_*r_x_ + C_y_r_y_*) (2)

While the relatedness term to self is unaffected by this, the relatedness terms to, for example, a full and a half-sibling (*r_x_* = 0.5 and *r_y_* = 0.25, respectively) can be weighted downward according to perceived (un)certainty in relatedness. For example, if the certainty regarding the full-sibling was 75% and the half-sibling was 95%, the perceived relatedness values would be 0.375 and 0.2375, respectively. In this way, we can more accurately model how the mind might perceive the costs of inbreeding and determine whether these costs track hypothetical decisions regarding sex with family members.

### The Current Study

We designed a study to investigate whether human motivations to avoid inbreeding reflect the following predictions derived from inclusive fitness theory: (i) the strength of opposition to an event of inbreeding is tightly knit to the inclusive fitness cost of inbreeding; (ii) inclusive fitness costs apply similarly to decisions regarding both 1st and 3rd party inbreeding; and (iii) that the perception of these costs should be influenced by subjectively perceived certainty in relatedness^1^.

## Materials and Methods

### Participants

A population-based sample of 2,353 individuals (1,588 women, 765 men; *M*_age_ = 33.9, *SD* = 9.2, range 18–57) living in Finland completed an on-line study (Albrecht et al., 2014). Participants were recruited by obtaining a random sample of addresses from the Central Registry of Finland, which contains information about all individuals residing in Finland. Invitation letters were sent to the sampled addresses. The invitation letter included information about the study and asked invitees to log on to a website, on which data were collected using a tailor-made solution provided by Delosis Psytools. The data collection had received ethical permission by the institutional review board at the Department of Psychology at Åbo Akademi University and informed consent was obtained from all participants. The data collection was carried out in accordance with the Declaration of Helsinki.

### Measures

**FIGURE 1:**
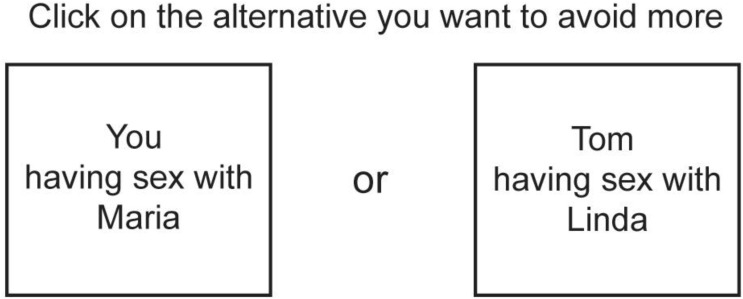
Example of a forced choice trial between two inbreeding scenarios. In this example, a male respondent is asked to choose between himself having sex with a female relative (i.e., direct inbreeding) and the respondent’s sibling having sex with a female relative (i.e., indirect inbreeding). Instead of terms for family-member categories (e.g., aunt, brother, or sister etc.), the names of the respondent’s actual relatives were used in the inbreeding scenarios.

Participants were first asked to report the number of relatives belonging to the categories of interest. If participants had more than one relative of a category (e.g., two half-siblings), subsequent questions concerned only one of these. Participants were at a later stage asked to make pairwise forced choices between different inbreeding scenarios selecting the alternative they wanted to avoid more. For each participant, the number of forced choices was limited to include only scenarios involving the participant’s own actual relatives. Only relatives older than 13 years of age were included in the scenarios. We used the names of their actual relatives in the scenarios presented. (see Figure 1 for an example of a trial).

**FIGURE 2:**
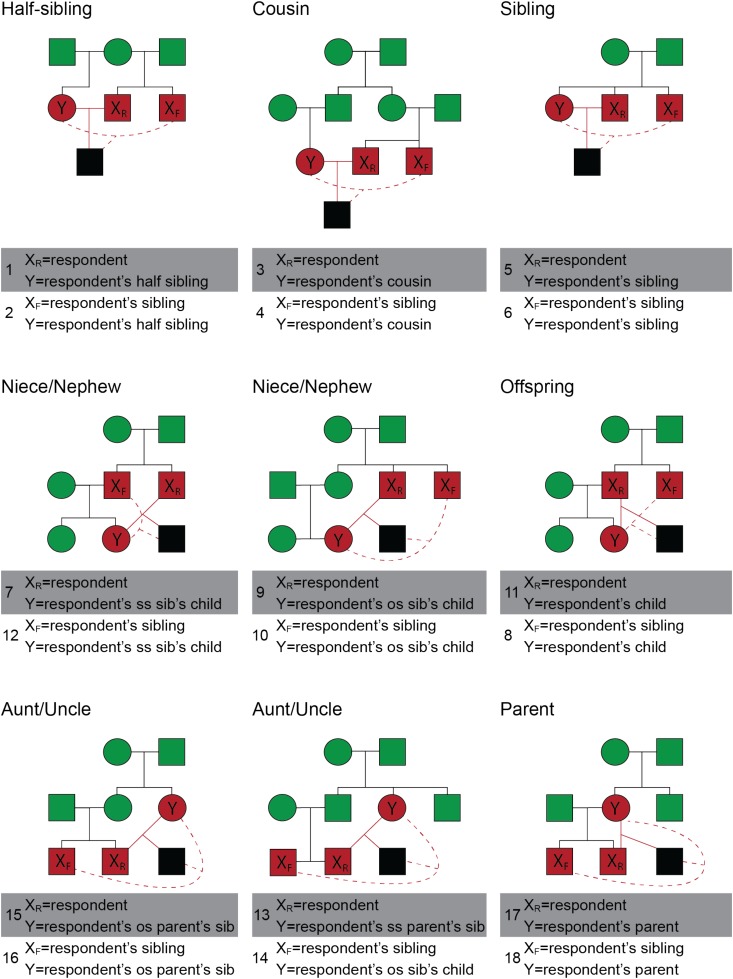
Pedigrees of the positions from which a respondent viewed various types of inbreeding in scenarios 1–18. The individuals engaging in inbreeding are displayed in red and their possible inbred offspring is displayed in black. Relatives of the inbreeding pair are displayed in green. Events of inbreeding are discussed as occurring between individual *X* and *Y*, where *X*_R_ is the respondent (participant), *X*_F_ is a family member of the respondent, and *Y* is a second family member. Inbreeding events that involved the respondent are shown with a solid red line; inbreeding events that involve two family members of the respondent are shown with a dashed red line. The top row of pedigrees illustrates sibling and cousin pairings; Row 2 depicts inbreeding events between respondents and younger family members (niece, nephew, child); Row 3 depicts inbreeding events between respondents and older family members (aunt, uncle, parent). Two events are depicted in each of the 9 pedigrees for a total of 18. As an example, the top left pedigree depicts two events: the respondent engaging in inbreeding with a half-sibling (scenario 1, solid red lines) and the respondent’s full sibling engaging in inbreeding with a half sibling (scenario 2, dashed red lines). SS indicates that the sibling or parent is of the same sex as the respondent and OS indicates that the sibling or parent is of the opposite sex. Squares refer to males and circles refer to females. All inbreeding scenarios are heterosexual and, for simplicity, only the male respondent versions are depicted.

There were two types of scenarios, direct and indirect inbreeding scenarios. Direct inbreeding scenarios included the participant and an opposite-sex relative, referred to by name. Indirect inbreeding scenarios included the participant’s same-sex sibling and an opposite-sex relative, both also referred to by name. All scenarios described heterosexual mating. Scenarios were selected to represent different levels of fitness costs, and to include both direct and indirect scenarios. A total of 18 possible inbreeding scenarios were included in the present study (Figure 2).

Because a scenario could not be paired with itself in a forced-choice situation, there were 153 possible combinations of the 18 inbreeding scenarios. As the study consisted of two separate data-collections (Albrecht et al., 2014)–one focusing on participants’ full siblings, half-siblings, cousins, as well as parents, uncles, and aunts, and one focusing on participants’ full siblings, half siblings, cousins, as well as children, nieces, and nephews–36 of the 153 possible pairings were unavailable. This means that the present study included a total of 117 possible pairings. To avoid fatigue effects and decrease the likelihood of random responding, we limited the number of choices to a maximum of 36 per participant. Because some relatives (e.g., half-siblings) were presumed to be less common than others (e.g., full-siblings) in the sampled population, a selection-order was predefined so that rare scenarios were preferentially displayed when possible (Table 1). Because participants were only presented with scenarios that included their actual relatives, the average number of choices made by each respondent (10.5, range 1–35) was lower than this. The order of the trials was randomized for each respondent. The total number of choices was 27,364. Of these, female respondents made 18,915 choices and male respondents made 8,449 choices.

**Table 1:** Inbreeding situations included in the pair-wise forced-choice paradigm and estimated fitness compromise.

Inbreeding situations	Preference order	Relatedness values				Fitness compromise
		*r_x_*	*r_y_*	*r_xy_*	δ*_xy_*	δ*_xy_*(*r_x_ + r_y_*)
1. Respondent & Respondent’s Half-sibling	2	1.000	0.250	0.250	0.150	0.188
2. Respondent’s sibling & Respondent’s Half-sibling	1	0.500	0.250	0.250	0.150	0.113
3. Respondent & Respondent’s Cousin	6	1.000	0.125	0.125	0.075	0.084
4. Respondent’s sibling & Respondent’s Cousin	5	0.500	0.125	0.125	0.075	0.047
5. Respondent & Respondent’s Sibling	4	1.000	0.500	0.500	0.300	0.450
6. Respondent’s sibling & Respondent’s Sibling	3	0.500	0.500	0.500	0.300	0.300
7. Respondent & Respondent’s (SS) Sibling’s Child	4	1.000	0.250	0.250	0.150	0.188
8. Respondent’s sibling & Respondent’s (SS) Sibling’s Child	3	0.500	0.250	0.500	0.300	0.225
9. Respondent & Respondent’s (OS) Sibling’s Child	2	1.000	0.250	0.250	0.150	0.188
10. Respondent’s sibling & Respondent’s (OS) Sibling’s Child	1	0.500	0.250	0.250	0.150	0.113
11. Respondent & Respondent’s Child	6	1.000	0.500	0.500	0.300	0.450
12. Respondent’s sibling & Respondent’s Child	5	0.500	0.500	0.500	0.300	0.300
13. Respondent & Respondent’s (SS) Parent’s Sibling	4	1.000	0.250	0.250	0.150	0.188
14. Respondent’s sibling & Respondent’s (SS) Parent’s Sibling	3	0.500	0.250	0.250	0.150	0.113
15. Respondent & Respondent’s (OS) Parent’s Sibling	2	1.000	0.250	0.250	0.150	0.188
16. Respondent’s sibling & Respondent’s (OS) Parent’s Sibling	1	0.500	0.250	0.250	0.150	0.113
17. Respondent & Respondent’s Parent	6	1.000	0.500	0.500	0.300	0.450
18. Respondent’s sibling & Respondent’s Parent	5	0.500	0.500	0.500	0.300	0.300

*Preference order refers to the pre-defined selection order of dyads to include if a respondent otherwise would have had more than 36 trials in total. *r_*x*_* = degree of relatedness between the respondent and the person *X* in the scenario; *r_*y*_* = degree of relatedness between the respondent and the person *Y* in the scenario; *r_*xy*_* = the relatedness between persons *X* and *Y* in a scenario; and δ_*xy*_ = denotes the fitness compromise in the inbred offspring depending on the relatedness between *X* and Y*.

The expression for estimating the average fitness costs of each scenario comprised three components. The three components were the degree of relatedness of the focal person to person *X* (*r_x_*) and *Y* (*r_y_*) in a scenario, and the degree of relatedness between person *X* and *Y* (*r_xy_*). We then combined these components into the following expression: δ*_xy_*(*r_x_ + r_y_*). Independently of whether the participant him/herself was or was not involved in the scenario, this expression weights δ by the degree of relatedness between the two individuals engaging in inbreeding. This then reflects the decreased biological fitness in an inbred child. The decreased biological fitness of this child is then weighted by its relatedness to the respondent via both of the two persons engaging in the inbreeding (e.g., the respondent him/herself and the respondent’s half-sister). We used a value 0.6 for δ, meaning that the average fitness compromise in offspring to inbreeding siblings or parents and their children (i.e., the highest possible level of inbreeding) would be 30% (Bittles, 1983; Bittles and Neel, 1994). Note that the absolute size of this value does not influence the relative difference between the costs of each scenario. The value only reflects a hypothetical value of the fitness decrease in inbreeding compared to breeding between persons that are not closely related. We, however, found it worthwhile to include a reasonable estimate for δ, since doing this results in a more meaningful value describing the estimated fitness cost. (see Table 1 for a list of possible inbreeding situations, relatedness information, and the estimated fitness costs from each scenario).

Perceived certainty in relatedness was measured by asking the participants to report, on a scale from 0 to 100 (not at all certain–very certain), how certain they were in the biological relationship to each of their actual relatives. Participants were not asked to report this for themselves, neither were mothers asked to report this with respect to biological children. In both cases, full certainty was assumed and values of 100 were imputed. The mean kinship certainty was 78.2 (*SD* = 37.2) and ranged from 0 to 100.

### Statistical Analysis

For analyses, we used Excel for Mac 16.11.1 and *R* 3.2.3 in R Studio 0.99.824. We conducted two types of statistical analyses. To analyze the overall probability that one scenario had been chosen over the other scenarios with which it had been compared, we first created a matrix with all possible comparisons presented as both rows and columns. In this matrix, values indicated how many times the row scenario was chosen as the more negative and how many times the column scenario was chosen as more negative. After this, the aggregated choice probabilities (the probability of a scenario being chosen over its alternative) could then be compared to the estimated fitness costs. This was done using a zero-order correlation (CORREL function in Excel) analysis between the aggregated choice probabilities and the fitness costs.

Because this aggregating approach could not effectively incorporate dyad-specific variables, such as the perceived certainty in relatedness to each of the described relatives, we also analyzed the data trial-wise. In this approach, each row in the long-format data described a choice that had been made. Each row also included the degree of relatedness between the respondent and the two individuals (i.e., *r_x_* and *r_y_*) in both of the two scenarios, and the relatedness between the two individuals included in the two scenarios (i.e., *r_xy_*). From these variables, the relative difference between the fitness compromises of the two scenarios in each trial was calculated as (δ*_xy_*(*r_x_ + r_y_*))_1_ – (δ*_xy_*(*r_x_ + r_y_*))_2_. To analyze whether this relative difference between fitness compromises explained the outcome of each choice, we used a multi-level binary logistic regression. To consider the clustering of choices within respondents, participant was added as a random intercept. This was done using the package lme4 (Bates et al., 2015) for *R*.

### Open Science Statement

All data used in the current manuscript and replicable scripts for analyses are available at the Open Science Framework (osf.io/pd7jb).

## Results

**FIGURE 3:**
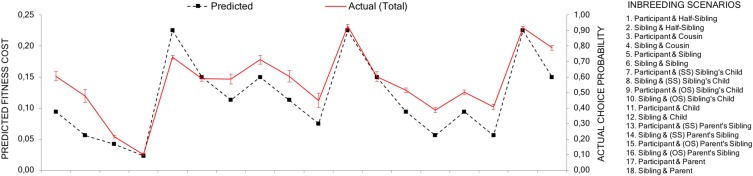
Predicted and actual avoidance. The black dotted line shows the predicted fitness costs of each scenario (1–18) for the respondent. The red line shows the actual avoidance values (the probability, i.e., proportions, a scenario had been chosen over its alternatives). Error bars represent 95% confidence intervals without continuity correction for proportions. The predicted and actual lines are placed on the same average level to make comparisons between then easier.

As predicted, the correlation between the aggregated choice probabilities and the estimated relative fitness loss between two scenarios was high (*r* = 0.883, *p* < 0.001). Although women have a stronger inbreeding aversion, the relative difference between different types of inbreeding scenarios should be similar for men and women. Indeed, the correlations were similar for male (*r* = 0.884, *p* < 0.001) and female respondents (*r* = 0.877, *p* < 0.001) (see Figure 3). This suggests that, as a general pattern, actual choices closely follow the effects that an event of inbreeding has on inclusive fitness (see Appendix Table A1 for a complete matrix of choice distribution).

We then structured the data trial-wise to test whether the difference between the inclusive fitness costs incurred by each of the two scenarios presented and whether perceived kinship certainty to the individuals in the scenarios predicted individual choices. The association between difference in fitness compromise and choice was statistically significant, *z* = -74.38, *p* < 0.001. Of the 27,364 choices, 83.6% were classified correctly based on the difference between the inclusive fitness costs of the two scenarios. Also in this case, classification accuracy was similar for men, 83.3% and women, 83.7%. When trials including scenarios with equal fitness costs were removed, classification accuracy was 85.2%.

To further test whether the choices followed a gene’s-eye perspective rather than being biased toward avoiding costs to self, we then grouped the trials in three separate groups. The first group consisted of all trials (*n* = 10,098) that included two 1^st^ person scenarios (e.g., “You having sex with Maria” vs. “You having sex with Linda”). The second group consisted of all trials (*n* = 4,295) that included two 3rd person scenarios (e.g., “Tom having sex with Maria” vs. “Tom having sex with Linda”). The third and final group consisted of all trials (*n* = 12,971) that included one 1st person and one 3rd person scenario (e.g., “You having sex with Maria” vs. “Tom having sex with Linda”). We added the term Type as a predictor alongside the term for the difference in fitness compromise. The association between difference in fitness compromise and choice remained statistically significant, *z* = -72.95, *p* < 0.001. With 1st vs. 1st trials as the reference category, the difference was statistically significant for 1st vs. 3rd trials, *z* = -12.33, *p* < 0.001, but not for 3rd vs. 3rd scenarios, *z* = 1.10, *p* = 0.271. The percentage of correctly classified choices in these three groups was 85.2% (1st vs. 1st), 84.2% (3rd vs. 3rd), and 82.6% (1st vs. 3rd).

We then investigated the effect of perceived certainty in relatedness. We first added the term for perceived certainty alongside the term for difference in fitness compromise. The association between difference in fitness compromise and choice remained statistically significant, *z* = -31.80, *p* < 0.001. The association between perceived certainty and choice was statistically significant, *z* = 9.44, *p* < 0.001. The classification accuracy was now 83.8%. Considering that the relatively high kinship certainty made it unlikely that variations in kinship certainty would overturn decisions in choices between scenarios with unequal fitness consequences, we then limited the analysis to the 2,323 trials where there was an equal theoretical fitness cost to both scenarios. In this case, we only included the term for perceived certainty. We found that 87.7% of these choices were correctly classified.

## Discussion

Analyzing data from a large population-based sample, we show that decisions regarding inbreeding closely follow predictions derived from inclusive fitness theory. We also show that these decisions–at least partly–are based on psychologically estimated relatedness. Although other factors (e.g., age differences, number of siblings, and the health and reproductive status of the relatives) that may affect these decisions were not considered, our theoretically derived predictions explained a large proportion of the forced-choice decisions respondents made with regards to which of two inbreeding situations to avoid. Critically, we demonstrate that there is little effect of whether the respondent him/herself was involved in the inbreeding scenario or not. Classification accuracy was high for all possible combinations of 1st vs. 3rd perspective inbreeding scenarios.

Although the current study does not provide insight into the actual mechanisms that regulate opposition to inbreeding (i.e., whether or not it is mediated by cultural norms, and if so, whether or not cultural norms are independent of psychological adaptations), there is a vast literature suggesting that mating between close kin (i.e., closer than first-degree cousins) is rare in most cultures (Thornhill, 1993; Wolf and Durham, 2004), and that this is true even when it is not explicitly discouraged by cultural norms. For example, data from Taiwan and the Middle East suggest that even when cultural norms encourage mating between individuals raised under sibling-like circumstances, environmental cues of relatedness negatively affect sexual attraction (Talmon, 1964; Wolf, 1968, 1970; McCabe, 1983). This is in line with the hypothesis of an adapted psychology that uses cues of kinship that have correlated with kinship in our evolutionary past, and that these kinship estimates regulate our kin-directed behavior (Westermarck, 1891; Lieberman et al., 2007; Antfolk, 2014). Our study extends this theory to include also opposition toward others engaging in inbreeding, with harmful inclusive fitness costs to a focal individual.

Perceived relatedness is also likely to be a function of both the type of biological relationship and the degree of relatedness. For example, whereas a woman can be almost certain a child born by her is a biological child, a man can almost never be fully certain he is the biological father (Tal and Lieberman, 2007). Also, younger siblings have to rely on other (and arguably less valid cues) of relatedness (i.e., co-residence), whereas older siblings can rely on maternal perinatal association (Lieberman et al., 2007). Moreover, each instance of paternity that separates two individuals (one for siblings, three for cousins through a paternal uncle) should decrease perceived relatedness. This means that degree of relatedness might be negatively related to kinship certainty.

Humans tend to react with disgust when asked to contemplate sex with close kin (Royzman et al., 2008; De Smet et al., 2014), and there is some prior evidence that the strength of this reaction reflects the fitness costs associated the degree of inbreeding (Fessler and Navarrete, 2003; Lieberman et al., 2003; Antfolk et al., 2012a, 2014a; Kresanov et al., 2018). There is also some evidence that this response is lacking in individuals (i.e., biological siblings) who have not experienced certain kinship cues (e.g., co-residence) in their childhood environment. In this case, sexual attraction might be present irrespectively of cultural norms condemning their sexual relationship. Examples of this can be found in legal cases where a sibling pair reared apart later have had children together (e.g., Stübing vs. Germany, 2012). Because cultural norms, reflected in legal definitions, often are relatively imprecise and do not, for example, condemn sex between full-siblings more strongly than sex between half-siblings (in most cases, both are equally punishable), the close fit between choices and fitness costs (e.g., more opposition to inbreeding between full siblings than to inbreeding between half-siblings) might not be fully explainable as the consequence of societal norms. It is also important to point out that all the study was conducted on an entirely Finnish sample. Because of this, the results of the current study should be generalized to other populations with caution. Studies that replicate the current methodology in different cultural settings could allow us to understand the role of cultural norms in shaping opposition to inbreeding.

Another limitation of the current study is that the method measures mate choices outside of its natural context. The findings should therefore not be taken as evidence of one type of inbreeding actually being more likely than another, although studies suggest this to be real possibility (Sariola and Uutela, 1996). Actual sexual behavior is influenced by a number of factors that are beyond the scope of the present study. Nevertheless, ethical considerations make it impossible to experimentally study the phenomenon of inbreeding avoidance *in vivo*.

## Conclusion

We conclude that humans perceive the indirect fitness costs of mating decisions made by close family members and that the strength of opposition against inbreeding are explainable in terms of inclusive fitness costs.

## Author Contributions

JA, PS, CH, and DL conceptualized and planned the study. JA, CH, and AA collected the data. JA, PS, CH, and AM analyzed the data. JA, PS, and DL wrote the manuscript. All authors provided their comments on the manuscript. JA and CH prepared figures and tables.

## Conflict of Interest Statement

The authors declare that the research was conducted in the absence of any commercial or financial relationships that could be construed as a potential conflict of interest.

## Footnotes

1 Two age values (89 and 99) were winsorized because they were outside the sampled age range.

## Appendix

**Table A1:** Comparisons between inbreeding scenarios included in the pair-wise forced-choice paradigm and actual choice distributions.

	1	2	3	4	5	6	7	8	9	10	11	12	13	14	15	16	17	18
1		83| 16	219| 38	80| 5	24| 66	15| 13	4| 8	3| 9	6| 5	0| 1	3| 42	4| 5	73| 34	32| 7	63| 33	18| 6	15| 120	7| 14
2			63| 22	75| 10	6| 22	9| 19	0| 12	1| 11	1| 1	0| 1	0| 10	3| 6	20| 21	27| 12	17| 15	21| 3	4| 24	3| 18
3				957| 288	113| 1181	86| 523	13| 151	19| 145	12| 130	10| 58	29| 305	17| 153	101| 789	147| 347	106| 753	123| 342	49| 959	35| 399
4					33| 576	50| 559	11| 153	12| 152	4| 68	7| 61	5| 168	5| 165	50| 446	58| 436	35| 441	42| 423	11| 442	11| 423
5						537| 114	58| 36	43| 51	94| 62	54| 19	15| 183	36| 41	435| 109	219| 34	427| 106	215| 30	75| 484	51| 88
6							48| 46	15| 79	31| 46	37| 36	5| 74	16| 61	182| 73	216| 37	169| 79	206| 39	14| 133	18| 121
7								52| 120	24| 24	35| 10	5| 75	44| 34	–	–	–	–	–	–
8									26| 19	39| 6	6| 73	46| 32	–	–	–	–	–	–
9										38| 5	10| 58	8| 7	–	–	–	–	–	–
10											2| 13	2| 13	–	–	–	–	–	–
11												168| 12	–	–	–	–	–	–
12													–	–	–	–	–	–
13														382| 134	325| 330	242| 115	53| 673	41| 255
14															118| 239	169| 188	11| 292	10| 286
15																223| 87	37| 665	35| 256
16																	13| 278	8| 283
17																		384/114
18																		

*In each cell, the first value indicates how many times the row scenario was chosen as more aversive than the column scenario. The second value indicates how many times the column scenario was chosen as more aversive than the row scenario*.
